# Gloved to Death

**Published:** 1857-02

**Authors:** 


### GLOVED TO DEATH.

There are many almost inappreciable sappers of our life, any one of which might be in operation for a long time without causing any alarming condition of the system ; but when a multitude of these are at work, critical symptoms appear with alarming rapidity. The purest water will become putrid, if allowed to stagnate. The purest air from the ocean or the poles, if kept still, becomes corrupt in the cleanliest habitation in the land, and the healthiest blood in the system begins in a moment to die, if for a moment it is arrested in its progress through the system. In either of these cases of fresh water, of pure air, and healthy blood, corruption is the inevitable result of stagnation. To keep them all pure and life-giving, activity of motion is a physical necessity. Whatever tends to arrest or impede the flow of the blood through the body, does in that same proportion inevitably engender disease ; any other result is physically impossible, because impure blood is the foundation or an attendant of all sickness.

Very recently, a New Yorker purchased a pair of boots, but they fitted so tightly that he was compelled to take them off before night, but they caused his death within forty-eight hours.

The most unobservant know that cold feet and hands are uniform symptoms in those diseases which gradually wear our lives away. The cause of these symptoms is a want of circulation. The blood does not pass to and from the extremities with facility. Nine-tenths of our women, at least in cities and large towns, have cold feet or hands, or both; hence, not one in a hundred is healthy. It is at our feet and hands that we begin to die, and last of all the heart, because, last of all, stagnation takes place there. In the worst cases of disease, the physician is hopeful of recovery, as long as he can keep the extremities warm: when that cannot be done, hope dies within him. It needs no argument to prove that a tight glove prevents the free circulation of blood through the hands and fingers. It so happens, that the very persons who ought to do everything possible to promote the circulation of the blood, are those who most cultivate tight gloves, to wit: the wives and daughters who have nothing to do but dress; or rather, do nothing but dress; or to be critically accurate, who spend more time in connection with dressing, than on all other objects together, not including sleep. No man or woman born has any right to do a deliberate injury to the body for a single hour in the day; but to do it day after day, for a lifetime, against the lights of science and common sense, is not wise. We may wink at it, glide over it, talk about this being a free country, that it is ridiculous for a doctor to dictate whether a glove shall be worn tight or loose, but the effect won't be laughed or scorned away, for whatever is done which impedes the circulation of the blood, is done wrongfully against our bodies, and will be as certain of injurious results, as the hindering of any law, physical or physiological. Every grain of sand must be taken care of, or the universe would dash to atoms; and so with the little things of the body.

